# Emerging Indications for Hyperbaric Oxygen Treatment: Registry Cohort Study

**DOI:** 10.2196/53821

**Published:** 2024-08-20

**Authors:** Hideaki L Tanaka, Judy R Rees, Ziyin Zhang, Judy A Ptak, Pamela M Hannigan, Elaine M Silverman, Janet L Peacock, Jay C Buckey, Multicenter Registry for Hyperbaric Oxygen Treatment Consortium

**Affiliations:** 1 Division of Undersea and Hyperbaric Medicine Department of Emergency Medicine University of California at San Diego San Diego, CA United States; 2 Geisel School of Medicine Dartmouth College Lebanon, NH United States; 3 Dartmouth-Hitchcock Medical Center Lebanon, NH United States; 4 see Acknowledgments

**Keywords:** hyperbaric oxygen, inflammatory bowel disease, calciphylaxis, post–COVID-19 condition, PCC, postacute sequelae of COVID-19, PASC, infected implanted hardware, hypospadias, frostbite, facial filler, pyoderma gangrenosum

## Abstract

**Background:**

Hyperbaric oxygen (HBO_2_) treatment is used across a range of medical specialties for a variety of applications, particularly where hypoxia and inflammation are important contributors. Because of its hypoxia-relieving and anti-inflammatory effects HBO_2_ may be useful for new indications not currently approved by the Undersea and Hyperbaric Medical Society. Identifying these new applications for HBO_2_ is difficult because individual centers may only treat a few cases and not track the outcomes consistently. The web-based International Multicenter Registry for Hyperbaric Oxygen Therapy captures prospective outcome data for patients treated with HBO_2_ therapy. These data can then be used to identify new potential applications for HBO_2_, which has relevance for a range of medical specialties.

**Objective:**

Although hyperbaric medicine has established indications, new ones continue to emerge. One objective of this registry study was to identify cases where HBO_2_ has been used for conditions falling outside of current Undersea and Hyperbaric Medical Society–approved indications and present outcome data for them.

**Methods:**

This descriptive study used data from a web-based, multicenter, international registry of patients treated with HBO_2_. Participating centers agree to collect data on all patients treated using standard outcome measures, and individual centers send deidentified data to the central registry. HBO_2_ treatment programs in the United States, the United Kingdom, and Australia participate. Demographic, outcome, complication, and treatment data, including pre- and posttreatment quality of life questionnaires (EQ-5D-5L) were collected for individuals referred for HBO_2_ treatment.

**Results:**

Out of 9726 patient entries, 378 (3.89%) individuals were treated for 45 emerging indications. Post–COVID-19 condition (PCC; also known as postacute sequelae of COVID-19; 149/378, 39.4%), ulcerative colitis (47/378, 12.4%), and Crohn disease (40/378, 10.6%) accounted for 62.4% (n=236) of the total cases. Calciphylaxis (20/378, 5.3%), frostbite (18/378, 4.8%), and peripheral vascular disease–related wounds (12/378, 3.2%) accounted for a further 13.2% (n=50). Patients with PCC reported significant improvement on the Neurobehavioral Symptom Inventory (NSI score: pretreatment=30.6; posttreatment=14.4; *P*<.001). Patients with Crohn disease reported significantly improved quality of life (EQ-5D score: pretreatment=53.8; posttreatment=68.8), and 5 (13%) reported closing a fistula. Patients with ulcerative colitis and complete pre- and post-HBO_2_ data reported improved quality of life and lower scores on a bowel questionnaire examining frequency, blood, pain, and urgency. A subset of patients with calciphylaxis and arterial ulcers also reported improvement.

**Conclusions:**

HBO_2_ is being used for a wide range of possible applications across various medical specialties for its hypoxia-relieving and anti-inflammatory effects. Results show statistically significant improvements in patient-reported outcomes for inflammatory bowel disease and PCC. HBO_2_ is also being used for frostbite, pyoderma gangrenosum, pterygium, hypospadias repair, and facial filler procedures. Other indications show evidence for improvement, and the case series for all indications is growing in the registry.

**International Registered Report Identifier (IRRID):**

RR2-10.2196/18857

## Introduction

### Background

Hypoxia and inflammation are part of the pathophysiology for various conditions across a range of medical subspecialties. One approach to relieving hypoxia and reducing inflammation is the use of hyperbaric oxygen (HBO_2_). HBO_2_ is 100% oxygen delivered at pressures >1.4 atmospheres absolute (ATA) within a pressurized chamber. Typically, pressures of ≥2.0 ATA are used. HBO_2_ treatments greatly increase the amount of oxygen dissolved in plasma and tissue during the treatment and are very effective for relieving hypoxia. The high levels of oxygen in tissue lead to a variety of biochemical effects including reduced inflammation and the release of stem cells from the bone marrow [1,2], leading to its application in several conditions, often in cases when standard treatment is not effective.

Currently, the Undersea and Hyperbaric Medical Society (UHMS) has identified 15 conditions where HBO_2_ can be considered an approved treatment [3]. These range from caisson disease (decompression illness), where the combination of increased pressure, relief of hypoxia, and reduced inflammation from HBO_2_ help combat the impaired circulation and endothelial damage caused by bubbles [4], to radiation injury, where the pulses of oxygen promote angiogenesis and wound healing [3]. Because of HBO_2_’s effects on hypoxia and inflammation, more medical diagnoses likely exist that can benefit from HBO_2_. However, HBO_2_ is typically given in long courses (20-40 treatments), and most centers see only a limited number of patients. Therefore, gathering outcome data on HBO_2_ treatment has been limited, and often, only case reports or small case series are available to support its use. In addition, practice patterns differ across centers, and some centers may use HBO_2_ successfully for an indication that other centers may not consider it for. To gather more data on HBO_2_ applications, outcome data from multiple centers need to be combined, but until 2011, no major academic registry existed to record treated cases and to track outcomes [5,6].

The International Registry for Hyperbaric Oxygen Treatment was formed to gather consistent outcome data from multiple centers [5]. The registry’s goal is to improve the use of HBO_2_ through evidence-based medicine. The registry records all hyperbaric medicine cases seen at participating centers and includes data on demographics, outcomes, complications, treatment duration, treatment pressure, and quality of life (QOL), among other markers. The registry started at the Dartmouth-Hitchcock Medical Center and Elliott Hospital in 2011 but expanded significantly in 2020 when the number and geographical distribution of participating centers grew. A total of 32 centers are entering data as of May 2024. The registry includes centers in the United States, the United Kingdom, and Australia (Table 1), and the data are used in publications about the use of HBO_2_ [6-8].

An important outcome from the registry is identifying emerging medical indications for HBO_2_ treatment. Particularly at academic centers, unique or challenging cases where hypoxia, inflammation, or both are considerations for referring patients for HBO_2_ treatment. The types of cases referred and the outcomes from them can indicate which new indications may need further study in controlled trials. This information can also support the use of HBO_2_ for individual conditions when other treatments are not effective. Since the registry’s inception, 378 cases have been recorded where treatment was given for a condition falling outside of the current 15 UHMS-approved indications.

**Table 1:** Center totals for emerging indications contributing to the analysis (n=378).

Center	Location	Total cases in registry (n=9019), n	Total emerging indications for HBO_2_ therapy (n=378), n (%)
Alfred Health	Melbourne, Australia	135	1 (0.3)
Avera McKennan Hospital	Sioux Falls, South Dakota	310	10 (2.6)
Beverly Hospital	Beverly, Massachusetts	67	2 (0.5)
DDRC^a^ Health Care	Plymouth, United Kingdom	109	4 (1.1)
Dartmouth-Hitchcock Medical Center	Lebanon, New Hampshire	1118	74 (19.6)
Duke University Medical Center	Raleigh, North Carolina	319	4 (1.1)
James Paget University Hospital	Great Yarmouth, United Kingdom	47	4 (1.1)
Intermountain Medical Center	St Murray, Utah	495	10 (2.6)
Latter Day Saints Hospital	Salt Lake City, Utah	283	2 (0.5)
Legacy Health Group	Portland, Oregon	645	12 (3.2)
LHM^b^ Health Care	London, United Kingdom	131	6 (1.6)
Logan Regional Medical Center	Logan, Utah	110	1 (0.3)
Mayo Clinic	Rochester, Minnesota	947	11 (2.9)
Midlands Diving Chamber	Rugby, United Kingdom	171	152 (40.2)
Prince of Wales Hospital	Sydney, Australia	847	36 (9.5)
Spectrum Health	Grand Rapids, Michigan	409	6 (1.6)
St George Regional Hospital	St George, Utah	310	5 (1.3)
St Richard’s Hospital	Chichester, United Kingdom	15	4 (1.1)
University of California San Diego	San Diego, California	385	12 (3.2)
University of Maryland Medical Center	Baltimore, Maryland	1260	29 (7.7)
University of Pennsylvania	Philadelphia, Pennsylvania	223	1 (0.3)
University of Rochester Medical Center	Rochester, New York	63	2 (0.5)
Utah Valley Hospital	Provo, Utah	450	6 (1.6)
Wesley Hyperbaric	Brisbane, Australia	233	4 (1.1)

^a^DDRC: Diving Diseases Research Center.

^b^LHM: London Hyperbaric Medicine.

### Objectives

The goal of this analysis is to: (1) quantify which non–UHMS-approved indications are being treated at registry centers and (2) provide outcomes for indications where sufficient cases exist. This analysis is important to: (1) identify indications deserving of further research, (2) inform both hyperbaric and other practitioners on new and emerging uses of HBO_2_, and (3) identify potential applications of HBO_2_ treatment across various medical and surgical specialties for challenging cases.

## Methods

### Overview

To guide the presentation of data in this report, the RECORD (Reporting of Studies Conducted Using Observational Routinely Collected Health Data) statement was used [9]. RECORD was created as an extension to the STROBE (Strengthening the Reporting of Observational Studies in Epidemiology) statement to address reporting items specific to observational studies using routinely collected health data such as registries. The checklist is included as supplemental material.

### Ethical Considerations

The structure of the web-based International Multicenter Registry for Hyperbaric Oxygen Therapy has been described previously [5]. Briefly, the registry is composed of hyperbaric centers that agree to follow the registry consortium agreement when becoming a member. Every site obtained institutional review board (IRB) approval and gathered informed consent in agreement with their IRB approval. Sites either obtain IRB approval from their own IRB or establish a reliance agreement with the IRB Dartmouth College (STUDY00024438). Informed consent differs across centers, with some centers having an approved waiver of consent and others requiring a separate consent from the hyperbaric treatment consent for all patients. Data are deidentified when they are sent to the data coordinating center. Participants do not receive compensation.

### Registry Design and Data Collection

All centers use a free database application, REDCap (Research Electronic Data Capture; Vanderbilt University) for data entry. REDCap is a secure, web-based software platform designed to support data capture for research studies, providing: (1) an intuitive interface for validated data capture, (2) audit trails for tracking data manipulation and export procedures, (3) automated export procedures for seamless data downloads to common statistical packages, and (4) procedures for data integration and interoperability with external sources. Registry data from each center are anonymized and collated every quarter at the coordinating center (Dartmouth) and are available to all users for research purposes [10,11].

Data are collected within a week of beginning and ending HBO_2_ treatment. All patients have relevant demographic data recorded (age, biological sex, and race). Yes or no questions are asked about any history of prior HBO_2_ treatment, prior seizures, or current pregnancy. Patients are asked about diabetes (whether controlled by diet, oral medications, or insulin) and about any current or prior smoking history or other nicotine use. At the end of treatment, the number of treatments given is recorded, along with any treatment complications that may have occurred.

Several patient-reported outcome questionnaires are used. All the questionnaires are maintained on the registry website. Any updates to documents are thus easily distributed to the centers that all have access. Patients are asked to complete the EuroQol EQ-5D-5L QOL questionnaire at the beginning and end of treatment [12]. This questionnaire asks about health in 5 dimensions: mobility, self-care, usual activities, pain or discomfort, and anxiety or depression. Each dimension has 5 levels, and the answers to the 5 questions are combined into 1 composite score or index. Scores range from –0.59 to 1, where 1 is the best possible reported health. Overall health is also rated on a 0 to 100 hash-marked, vertical visual analog scale (VAS). The EQ-5D-5L questionnaire is a copyrighted evidence-based assessment of health and has been registered for registry use. It has been incorporated into all entries collected since 2019 to evaluate overall health pre- and post-HBO_2_ therapy. Patients treated before then do not have results for this questionnaire.

Depending on the indication, additional questionnaires (eg, urinary distress inventory for radiation cystitis, European Organisation for Research and Treatment of Cancer, Quality of Life Questionnaire, Head and Neck 35 QLQ-H&N35 for head and neck cancer) are administered in accordance with registry protocols. For patients with Crohn disease, the perianal Crohn symptom index is completed, which asks about the number of fistulas, fistula drainage, rectal drainage, bowel movement frequency, and the use of motility agents. The administration of this questionnaire began in 2019, and it is included in Multimedia Appendix 1. Patients with ulcerative colitis (UC) receive a questionnaire that asks about bowel movement frequency during the night and day, blood in the stool, urgency, and pain. The frequency, blood, urgency, and pain factors are combined into an overall score that ranges from 0 (no symptoms) to 17 (maximum symptoms) to characterize symptom severity. The administration of this questionnaire also started in 2019, and it is included in Multimedia Appendix 2. Because the Crohn and UC questionnaires were added later in the registry’s development, patients treated before that time do not have results from these questionnaires.

**Figure 1:**
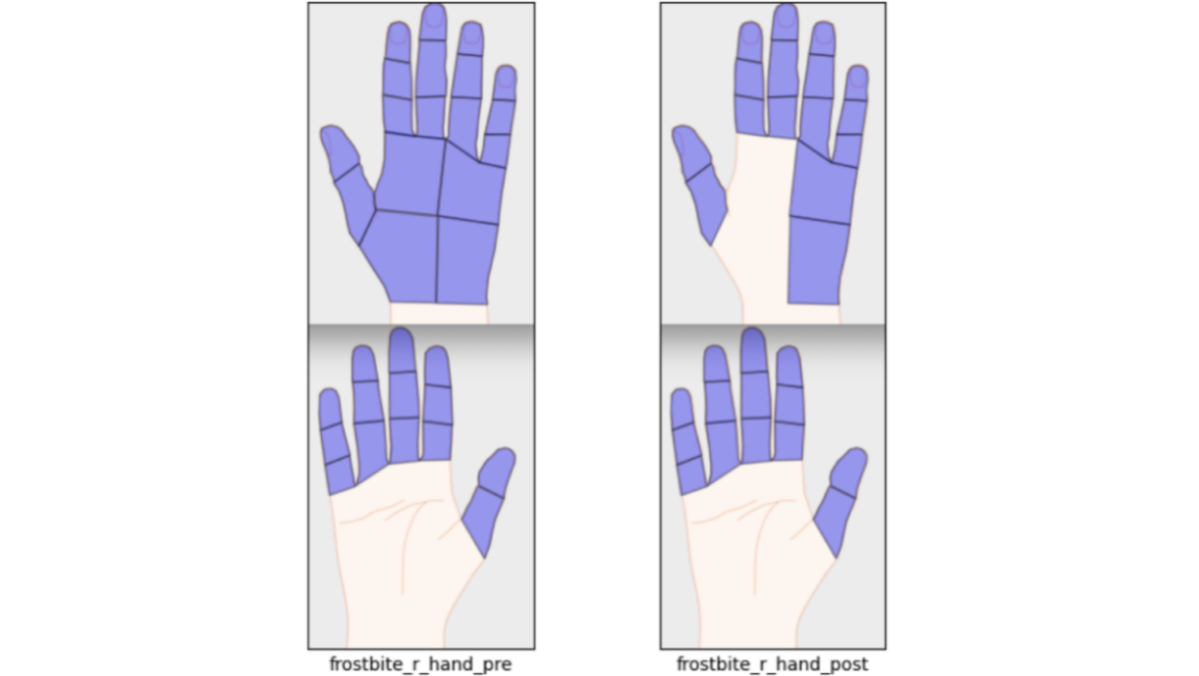
Data entry screen for frostbite injuries. The registry provides different approaches to data entry to make data entry easy and consistent.

Patients with head trauma and post–COVID-19 symptoms are presented with the Neurobehavioral Symptom Inventory (NSI) [13]. The NSI has 22 questions designed to cover symptoms related to concussion. Patients rate the severity of common symptoms such as headache, fatigue, and nausea on a scale ranging from 0 (none) to 4 (very severe), with the maximum total score of 88. Initially, this questionnaire was only administered to patients with carbon monoxide poisoning, but in 2022, this questionnaire was expanded to any brain-related condition, including those with post–COVID-19 condition (PCC; also known as postacute sequalae of COVID-19). For frostbite, the users are presented with a diagram of the hands and feet, and they select the affected areas (Figure 1).

As the data are coded by indication, records are classified into one or more of the 15 approved UHMS indications or categorized as “other.” The design of this study was to focus on all those records where the indication was “other” and to combine data for those indications. Data entries fitting into the “other” category were isolated and subsequently further divided into subgroups for investigation in this study. Data were reviewed manually to determine if the record fit into one of the already recognized “other” indications or if it was an indication that did not exist within the registry. For example, Crohn disease is a selection on the “Other Indications” dropdown menu; however, this option was not always present in the registry. Previously, users would select “other” and then enter Crohn disease in a text box. So, these text entries were examined to ensure the cases were properly classified. Once the text had been identified, data were processed using MATLAB 2023a (The MathWorks Inc) using text-recognition algorithms to classify the reasons for treatment properly. These results were manually double-checked for accuracy using Microsoft Excel. The study size was determined by the number of entries for “other” indications in the registry from registry inception in 2011 through May 2024.

### Missing Data Reasons

Either pre- or posttreatment data were missing for a variety of reasons. For some of the questionnaires, data collection did not start until the registry had been underway for several years. This is true for the EQ-5D-5L and the Crohn, UC, and NSI questionnaires. So, patients treated early in the registry did not have results for these questionnaires. In other cases, data were missing because the patient discontinued treatment, was discharged from the hospital, or transferred to another facility and was not available for the final questionnaire. Some patients were too ill to complete the questionnaires.

### Statistical Analysis

For those indications with sufficient responses (n≥6) and complete pre- and posttreatment data for the variable in question, pre- and posttreatment values were compared statistically. Questionnaire responses or rating scales where the answers were categories were analyzed using the sign test (eg, EQ-5D-5L, NSI, and bowel questionnaire). Otherwise, data were compared using the Wilcoxon signed-rank test (eg, fistula number on the Crohn symptom index). Individuals who were missing a pre- or posttreatment value for a variable were not included in the primary analysis.

### Treatment of Missing Data

The baseline characteristics of those with complete and incomplete data on Crohn-, UC-, and PCC-related questionnaires were calculated and presented in the results. We conducted sensitivity analyses to tease out the potential impacts of missing data as follows. Individuals who were missing EQ-5D questionnaire responses but had other data in the registry indicating they had a positive response to treatment were given pre- and posttreatment values based on the pre- and posttreatment mean values of those with complete data. For the remainder, we used best, worst, and average case scenarios. In the best case, we assumed all those with missing data improved to the same degree on average as those with complete data. For the worst case, we assumed all those with missing data worsened by the same average percentage that those with complete data improved. For the average case, we calculated the proportion of individuals with complete data that improved and applied that to the missing data. We took the view that sensitivity analyses were not appropriate when the number of cases with complete data was very small, and we used 10 as the limit, accepting that this is an arbitrary limit.

## Results

### Overview

As of May 2024, a total of 32 sites were actively entering data in the registry, and 24 (75%) of these centers contributed to the indications labeled “other” (Table 1). In total, 378 cases were marked as having diagnoses not currently UHMS approved and receiving at least 1 HBO_2_ treatment. One center had an interest in PCC and contributed 141 (94.6%) out of 149 cases for that indication. Aside from PCC, UC was the most common condition (n=47, 12.4%), followed closely by Crohn disease (n=40, 10.6%). Combined, inflammatory bowel disease (IBD; n=87) accounted for 23% of hyperbaric cases treated as “other” (n=378). If combined with pouchitis (n=1) and pyoderma gangrenosum (n=7), which are both often associated with IBD, total IBD and related cases accounted for 25.1% (95/378) of cases treated. Of the remaining diagnoses treated, calciphylaxis (n=20, 5.3%), frostbite (n=18, 4.8%), and peripheral vascular disease ulcers (n=12, 3.2%) each had ≥10 cases. There were 23 diagnoses with only a single case (Table 2). The result of this was a series of case cohorts and individual case studies gathered into a single database.

**Table 2:** Summary of other indications (n=378)^a^.

Indication	Values, n (%)
Post–COVID-19 condition	149 (39.4)
Ulcerative colitis	47 (12.4)
Crohn disease	40 (10.6)
Calciphylaxis	20 (5.3)
Frostbite	18 (4.8)
Peripheral vascular disease ulcer	12 (3.2)
Acute COVID-19	9 (2.4)
Pyoderma gangrenosum	7 (1.9)
Pterygium	7 (1.9)
Hypospadias	7 (1.9)
Osteonecrosis and avascular necrosis	6 (1.6)
Head trauma	5 (1.3)
Infected implanted hardware	5 (1.3)
Pneumatosis intestinalis	4 (1.1)
Facial filler	4 (1.1)
Ischemic bowel	3 (8)
Raynaud syndrome	2 (0.5)
Malignant otitis externa	2 (0.5)
Nonarteritic anterior ischemic optic neuropathy	2 (0.5)
Central retinal vein occlusion	2 (0.5)
Cyclophosphamide cystitis	2 (0.5)
Femoral head necrosis	2 (0.5)
Invasive fungal infection	1 (0.3)
Chronic anal fissure	1 (0.3)
Vasculitic ulcer	1 (0.3)
BK^b^ virus cystitis	1 (0.3)
Levamisole vasculitis	1 (0.3)
Graft-vs-host disease	1 (0.3)
Decubitus ulcer	1 (0.3)
Greater trochanteric pain syndrome	1 (0.3)
Rectovaginal fistula	1 (0.3)
Argon poisoning	1 (0.3)
Pouchitis	1 (0.3)
Chemotherapy-related bladder ulcer	1 (0.3)
After surgery in irradiated tissue	1 (0.3)
Recurrent perianal abscess	1 (0.3)
Tinnitus	1 (0.3)
Clostridium enterocolitis	1 (0.3)
Ligament and cartilage injury	1 (0.3)
Branch retinal artery occlusion	1 (0.3)
Axonotmesis	1 (0.3)
Nonhealing bowel anastomosis	1 (0.3)
Multiple sclerosis	1 (0.3)
Inclusion body myositis	1 (0.3)
Epidermolysis bullosa	1 (0.3)

^a^Some of the indications were case series with several cases; others were case reports of individual cases.

^b^BK: human polyomavirus 1.

### IBD and Related Conditions

#### Crohn Disease

**Figure 2:**
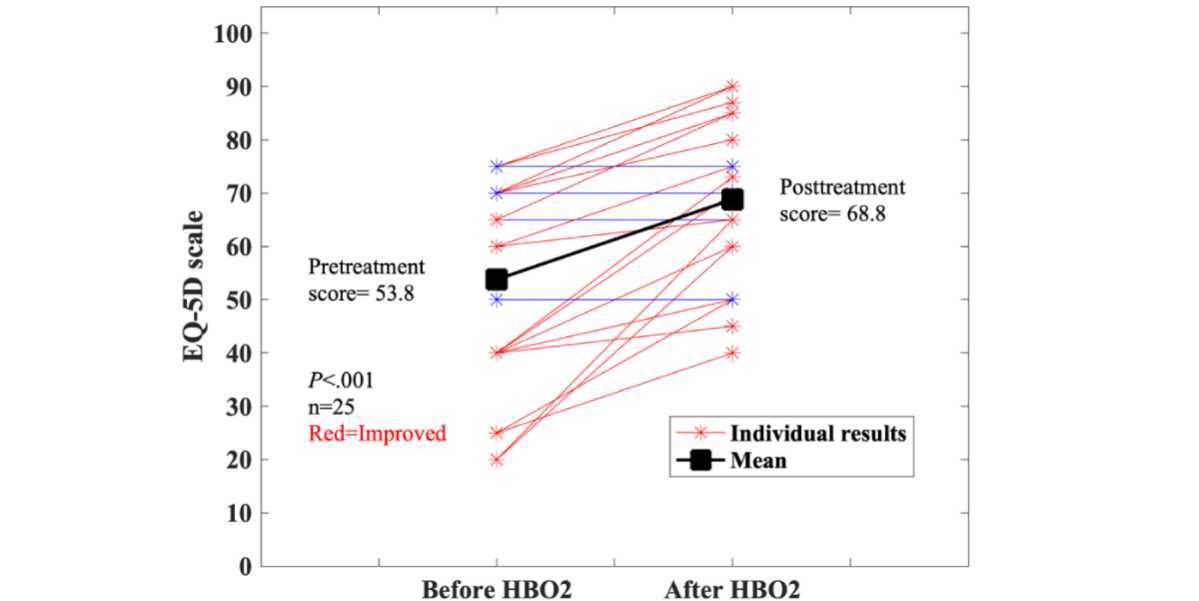
Results from the EQ-5D visual analog scale for patients with Crohn disease. A total of 20 patients had complete pretreatment and posttreatment hyperbaric oxygen (HBO2) data. A sensitivity analysis using a best, worst, and average case for missing data shows that the results are significant in all those cases.

**Figure 3:**
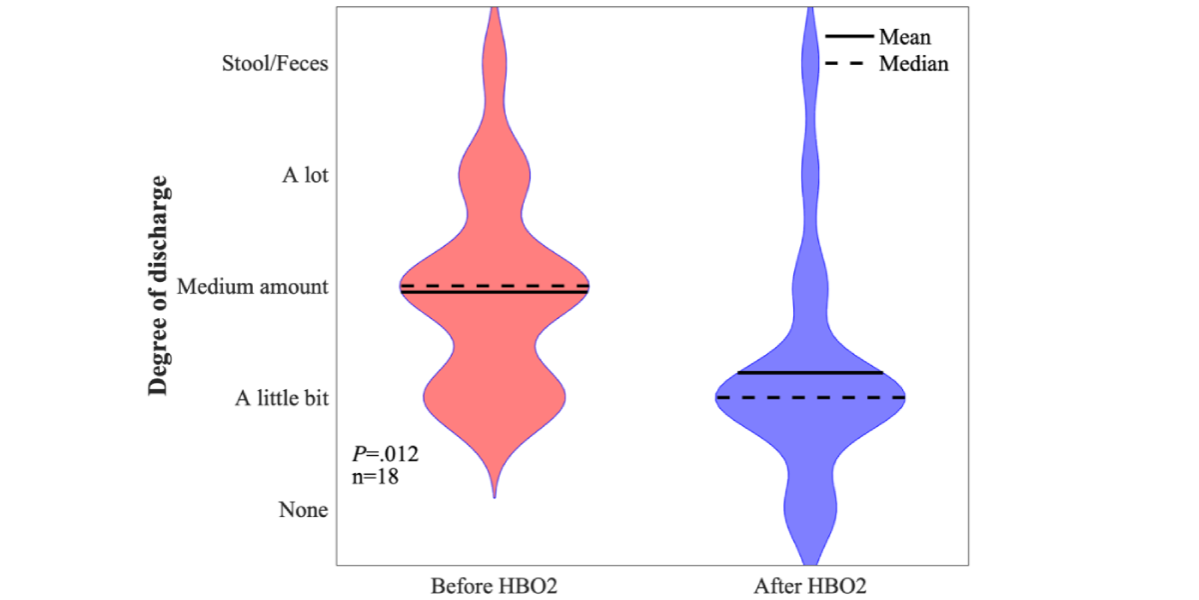
Reported fistula discharge pre- and post-HBO2 therapy in patients with Crohn disease who had complete pre- and post-HBO2 data. The sensitivity analyses results are follows: best case (25/33, 76% improved; *P*<.001), worst case 10/33, 30% improved; *P*=.33), and average case (18/33, 55% improved; *P*=.08).

The 40 patients with Crohn disease had a mean age of 37 (SD 13) years, with 14 (35%) men and 26 (65%) women. The racial breakdown was as follows: Asian (n=2, 5%); Black (n=2, 5%), White (n=28, 70%), more than 1 race (n=1, 2%), and missing (n=7, 18%). The median number of HBO_2_ treatments completed was 30 (IQR 19.5-40.5). A total of 25 (62%) of the 40 patients with Crohn disease had complete pre- and posttreatment data for the EQ-5D-5L QOL measure; 7 (18%) patients were missing data because they had been entered into the registry before the adoption of the questionnaire; 3 (8%) did not complete the pretreatment questionnaire, and an additional 5 (12%) did not complete the posttreatment questionnaire. Of the 3 with no pretreatment questionnaire, 1 transferred to another HBO_2_ center and 1 was reported to have improved. Of those 5 with pretreatment data but no posttreatment questionnaire data, 1 was improving, but insurance would not approve additional treatments, 1 had closed a fistula and stopped treatment, and 1 was discharged from the hospital and could not continue as an outpatient. One completed 6 treatments and was feeling too sick to continue. Of those who completed the EQ-5D (n=25), 20 (80%) reported improvement and 5 (20%) were unchanged (*P*<.001; Figure 2). Patients with perianal Crohn questionnaire results (n=18) reported a significant decrease in discharge from the fistulas after treatment (*P*=.01; Figure 3); 5 (28%) patients also reported a decrease in the number of fistulas.

For the sensitivity analysis, the 7 individuals with missing EQ-5D questionnaires were slightly younger (32 vs 37 years) with a similar total number of treatments (n=40 vs n=37) and were considered to be missing at random. Of the remaining 8, a total of 3 (38%) were reported to have improved elsewhere in the registry and so were “given” values based on the pre- and posttreatment mean values of those with complete data, and 1 (12%) was reported to be too sick to continue, so was assumed to have worsened by the same average percentage that others had improved (28% in this case). For the remaining 4 (50%), the best, worst, and average case scenarios were used. The best case assumed that all the 4 individuals improved on the EQ-5D VAS by the average amount, and the worst case assumed that they worsened by the same average percentage that others had improved (28% in this case). For the average case, the same proportion of improvement for those with data (20/25, 80%) was applied to the 4 cases (ie, 3 improved and 1 worsened). These gave the best case (27/33, 82% improved; *P*<.001), worst case (23/33, 70% improved; *P*=.001), and average case (26/33, 79% improved; *P*<.001). If the 3 individuals with missing EQ-5D data, but were noted to improve, were assumed to have worsened instead, this would still have been statistically significant (*P*=.04).

For the fistula discharge data (n=18), there were 18 (45%) individuals with complete data. For the sensitivity analysis, the 3 (7.5%) individuals who reported improvement elsewhere in the registry were treated the same as the others with missing data. The sensitivity analyses results are as follows: best (25/33, 76% improved; *P*<.001), worst (10/33, 30% improved; *P*=.33), and average (18/33, 55% improved; *P*=.08).

#### HBO2 for UC

**Figure 4:**
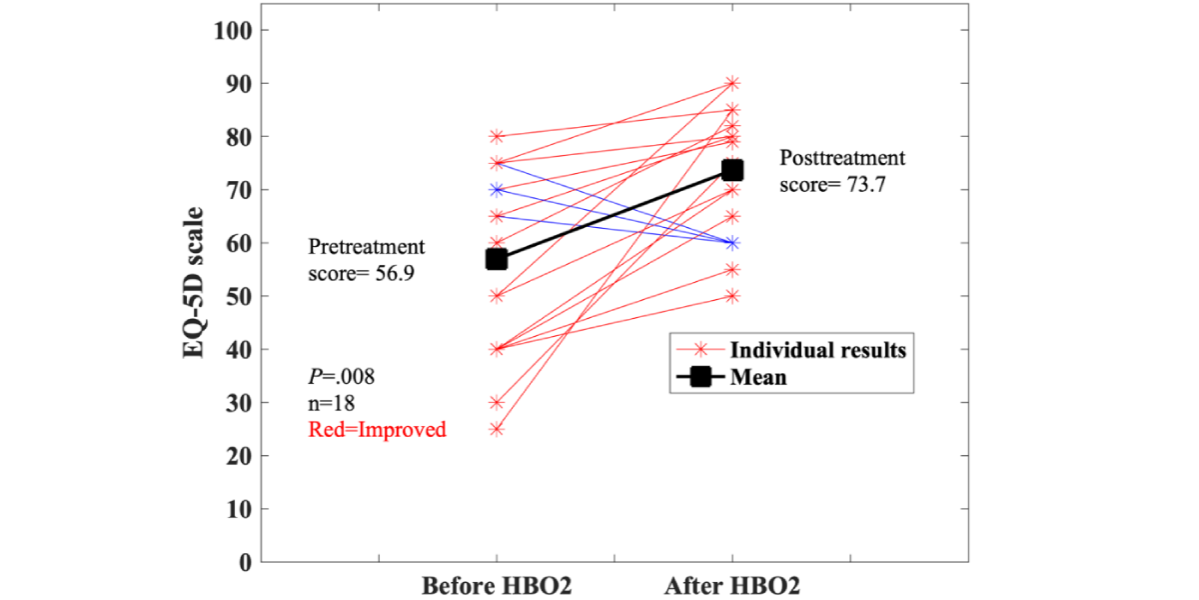
Results on the EQ-5D visual analog scale for patients with ulcerative colitis (UC) who had complete pre- and posthyperbaric oxygen (HBO2) data. The sensitivity analyses results are follows: best case (38/41, 93% improved; *P*<.001), worst case (19/41, 46% improved; *P*=.76), and average case (31/41, 76% improved; *P*=.002).

**Figure 5:**
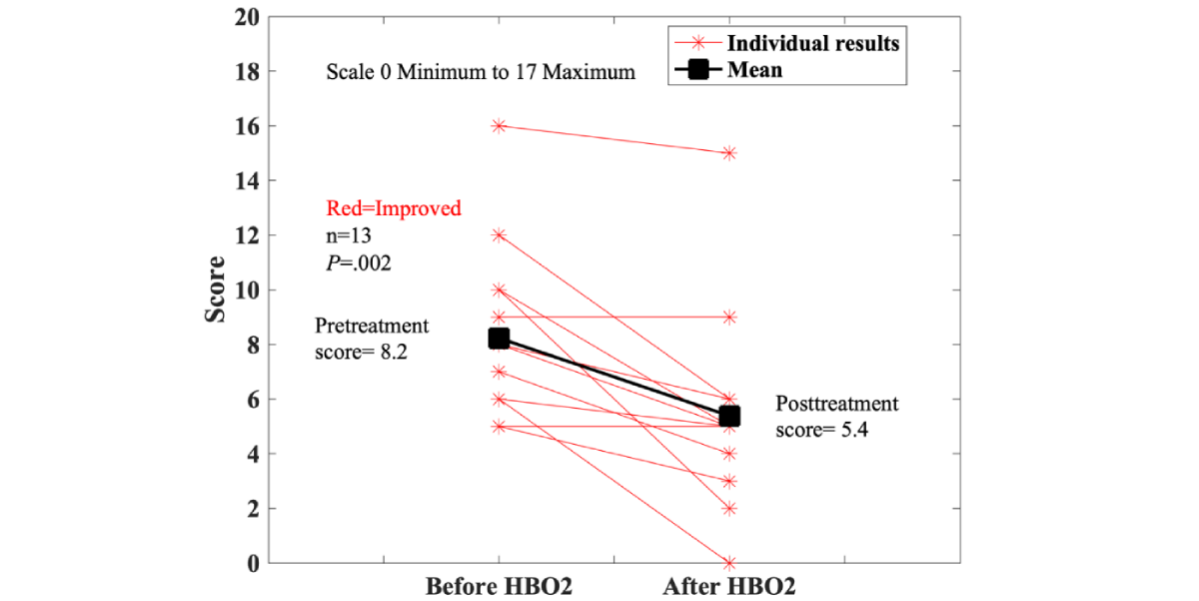
Results on the bowel questionnaire from the subset of patients with ulcerative colitis (UC) who had complete pre- and posthyperbaric oxygen (HBO2) data. The sensitivity analysis showed the best (38/41, 93%; *P*<.001) and worst (19/41, 46%; *P*=.14) cases, indicating that the results are sensitive to missing data.

The 47 patients with UC had a mean age of 41 (SD 20.5) years, with 24 (51%) women and 23 (49%) men. The racial breakdown was as follows: Asian (n=1, 2%); Black (n=2, 4%); White (n=40, 85%); refused (n=1, 2%); and missing (n=3, 6%). The median number of treatments was 5 (IQR 2.6-7.4). A total of 19 (40%) patients had complete EQ-5D-5L questionnaire data; 6 (13%) were missing data because they were in the registry before the EQ-5D was adopted; 12 (26%) had no pretreatment questionnaire data; and an additional 10 (21%) had no posttreatment questionnaire data. Of those with no pretreatment questionnaire data (n=12), 1 had cognitive impairment, 1 was unable to complete the form, 2 declined, 1 decided to stop treatment, 1 stopped because of an unrelated medical problem, and 4 stopped because they had improved. For 2 the reason was not listed. Among those with no posttreatment questionnaire data (n=10) despite having a pretreatment result, 4 (40%) were discharged before completing the form; in 3 (30%) cases, the patient terminated treatment; and in an additional 3 (30%), treatment ended unexpectedly. The group without pretreatment questionnaire data tended to be older (mean 43 years, SD 23.2) and receive more treatments (median 8.5, IQR 5.3-11.8). The group missing posttreatment questionnaire data had a similar mean age (40 years, SD 17.7) and median number of treatments (4.5, IQR 2-7) to the group having the questionnaire data. Of the 19 patients with UC who completed the EQ-5D, 3 (16%) had slightly decreased QOL scores, while most (n=16, 84.2%) had improvement (*P*=.008; Figure 4). The patients with UC who had questionnaire results also reported lower (better) scores on the bowel questionnaire (Figure 5). For the sensitivity analyses, the 4 UC cases that reported improvement were assumed to have improved by the mean of those with data. For the remainder, data are as follows: best case (38/41, 93% improved; *P*<.001), worst case (19/41, 46% improved; *P*=.76), and average case (31/41, 76% improved; *P*=.002).

There were 7 individuals with pyoderma gangrenosum, 6 (86%) women and 1 (14%) man with a mean age of 46 (SD 20.6) years. The racial breakdown was as follows: Asian (n=1, 14.3%); Black (n=1, 14.3%); and White (n=5, 71.4%). The median number of treatments was 26 (IQR 17.4-34.6). The data for these cases are limited because standard wound measures for these cases were implemented late in the registry. Only 2 (29%) cases had EQ-5D measurements; 2 (29%) cases were entered in the registry before beginning EQ-5D assessments; 2 (29%) had no pretreatment value; and 1 (14%) had no posttreatment value. A total of 3 (60%) had subjective data on improvement, and those 3 were reported to improve. One (20%) patient stopped due to an unrelated medical problem, and another (n=1, 20%) decided to stop after 7 treatments. The registry also includes 1 patient with pouchitis who improved.

#### Calciphylaxis

Calciphylaxis was the fourth most common “other” diagnosis (n=20; Table 3). The mean age was 61 (SD 15.9), with 12 (60%) women and 8 (40%) men. The racial breakdown was as follows: Black (n=2, 10%); White (n=15, 75%); refused (n=1, 5%); and missing (n=2, 100%). The median number of treatments was 27.5 (IQR 14.5-40.5). Only 3 (15%) patients had complete QOL data, while 5 (25%) individuals did not have EQ-5D data because they were treated before the EQ-5D was in the registry, 6 (30%) had no pretreatment questionnaire, and 6 (30%) had no posttreatment questionnaire. Of the 20 patients, 4 (20%) discontinued treatment, as they were too sick to continue, and 2 of these patients died. While 2 (10%) patients healed their wounds, 5 (25%) showed notable improvement. For those who improved, the median number of treatment sessions was 39 (IQR 31.1-46.9). The median number of hyperbaric treatments in the remaining cases was 21 (IQR 7.9-34.1). Sensitivity analyses were not used because the most useful outcome for this condition is improvement versus no improvement because of the high mortality. Many of the patients missing data either died or were seriously ill.

**Table 3:** Outcomes for the patients with calciphylaxis.

Total HBO_2_^a^ treatments		Original size (cm), L×W×D	Decrease length (%)	Decrease width (%)	Decrease depth (%)	Pretreatment EQ-5D VAS^b^	Posttreatment EQ-5D VAS	Reason for missing EQ-5D	Notes on outcome
**Improved**									
	8	12×10×4	33.3	30	75	—^c^	—	Before EQ-5D in registry	Patient decided to stop; better
	32	—	—	—	—	—	—	Not done	Improved
	29	7×1×0.5	98.6	80	100	—	—	Before EQ-5D in registry	Wounds healed
	18	11.3×5.4×2.5	—	—	—	50	40	—	Wounds improving; stopped due to other health issues
	40	45×15×25	8.9	30	0	30	—	Patient unable to return form	Despite size, considerably improved (granulation)
	60	3.7×5×0.1	67.6	–22	10	87	92	—	Wounds improved
	39	4.3×0.8×0.1	100	100	100	—	—	Before EQ-5D in registry	Transferred to other hospital, wounds healed
	48	L 1.9×5.0×2.2; R 2.0×9.5×2.2	58; 40	40; 26	82; 82	—	—	Before EQ-5D in registry	Wounds mildly improved
	40	—	—	—	—	30	80	—	Improvement in quality of life
**Not improved**									
	17	—	—	—	—	—	—	Unable	No change (wound care)
	11	5×2.5×1	–500	–500	–100	10	—	Patient did not return	Worsening or poor nutrition; stopped as futile
	21	—	—	—	—	30	—	Unable to complete	Worsening. Too sick to continue
	9	—	—	—	—	10	—	Stopped care	Patient decided to stop; claustrophobia noted
	21	110×100×5	—	—	—	—	—	Unable	No change
	26	7.1×4.9×3.5	—	—	—	50	—	Patient died	Transferred from outside hospital; died
	3	8×5×2	—	—	—	—	—	Declined	Patient decided to stop (nonmedical reason)
	10	—	—	—	—	—	—	Before EQ-5D in registry	Developed respiratory illness, died
	40	6×5×0	0	–10				Not done	Wounds unchanged per measurements
	40	—	—	—	—	—	—	Declined	No improvement
	40	—	—	—	—	40	—	Not done	No outcomes documented

^a^HBO_2_: hyperbaric oxygen.

^b^VAS: visual analog scale.

^c^Not available.

#### Frostbite

Frostbite was the fifth most common “other” diagnosis with 18 patients (Table 4). The mean age was 49 (SD 13.7) years, with 17 (94%) men and 1 (6%) woman. The racial breakdown was as follows: American Indian or Alaska Native (n=2, 11%); Black (n=1, 6%); White (n=12, 67%); other (n=2, 11%); and missing (n=1, 6%). The median number of treatments was 9 (IQR 7-11). Only 1 (6%) individual did not have EQ-5D data because the treatment occurred before the EQ-5D was in the registry, while 6 (33%) had no pretreatment questionnaire and 6 (33%) had no posttreatment questionnaire. For 2, the questionnaire was not done due to unspecified reasons; 3 either left treatment or refused treatment before completing the questionnaire; 1 had a language barrier; and 1 was not done due to inadequate staffing. For the 11 patients who had complete EQ-5D VAS data, there was a significant improvement (pretreatment score=48.2, posttreatment score=67.5; 9/11, 82% improved; *P*=.02). The sensitivity analyses results are as follows: the best case (16/18, 89% improved; *P*<.001), worst case (9/18, 50% improved; *P*=1.0), and average case (15/18, 83% improved; *P*=.002).

**Table 4:** Outcomes for patients treated for frostbite^a^.

Total HBO_2_^b^ treatments			Pretreatment EQ-5D VAS^c^		Posttreatment EQ-5D VAS		Pretreatment EQ-5D score		Posttreatment EQ-5D score		Frostbite classification		Reason for missing EQ-5D		Notes on outcome
**Improved**															
	7	10		60		0.16		0.084		3		—^d^		Improved, but had amputations	
	9	30		80		0.39		0.64		2		—		No amputations, improved	
	10	10				0.51				2		Not done		No amputations	
	4	85		95		0.33		0.44		4		—		Initial bilateral transmetatarsal amputations, subsequently improved	
	27	80		95								Only VAS		Had a skin graft	
	9	30		75		0.69		0.27		3		—		No amputations, good healing	
	10	45		48		0.53		0.37		2		—		Wounds stabilized	
	7	50		50		0.812		1		2		—		No amputations, wounds improved	
	10	80		50		0.40		0.42		2		—		Partial improvement, delay in HBO_2_ due to detox	
	7	50		70		0.41		0.80		2		—		No amputations, wounds improved	
	9	50		70		0.82		0.82		2		—		Wounds improved	
	10	20		50		0.06		0.26		3		—		—	
**Not improved or insufficient data**															
	30	—		—		—		—		2		Before EQ-5D in registry		Frostbite of hands, insufficient outcome data entered	
	15	0		—		–0.074				3		Had amputations, left treatment		Amputations on right and left foot. Homeless or drug use	
	4	—		—		—		—		—		Not done		Bilateral below-the-knee amputations	
	4	—		—		—		—		3		Language barrier		Behavioral issues or refused treatment. Bilateral below-the-knee amputations	
	6	—		—		—		—		—		Staffing		Psychiatric issues or refused treatment	
	2	75		—		0.58		—		—		Did not return		Left treatment	

^a^The frostbite classification scale: 1=first degree; 2=second degree; 3=third degree, 4=fourth degree (includes necrosis).

^b^HBO_2_: hyperbaric oxygen.

^c^VAS: visual analog scale.

^d^Not available.

### Peripheral Vascular Disease–Related Wounds

The sixth most commonly treated diagnosis was peripheral vascular disease–related wounds (Table 5). The mean age was 63 (SD 19.1) years, with 8 men and 4 women. The racial breakdown was as follows: 8 White, 2 Black, and 2 missing. The median number of treatments was 19.5 (IQR 8.3-30.8). Twelve cases were recorded, all involving arterial disease, but some also with concurrent venous disease. All wounds involved the lower extremities. Of those patients who had ≥20 HBO_2_ sessions, 5 of 6 improved, with the remaining 1 lacking enough data to reach a conclusion. The remaining 6 cases had ≤15 sessions. Four of these were worse, with 2 not having sufficient data recorded. The main outcome recorded was improved versus not improved or insufficient data, and there were insufficient EQ-5D data to do a sensitivity analysis.

**Table 5:** Outcome for patients with peripheral vascular disease.

Total HBO_2_^a^ treatments		Decrease in length (%)	Decrease in width (%)	Decrease in depth (%)	EQ-5D VAS^b^	EQ-5D VAS	EQ-5D score	EQ-5D score	Reason for missing EQ-5D	Notes on outcome
**Improved**										
	30	71.4	50	0	—^c^	60	—	—	Not given pre	Improved or healing
	30	59.7	47.3	0	85	90	0.83	1.0	—	Improved or healing
	30	66.7	83.3	83.3	70	70	0.68	0.62	—	Improved or healing
	24	100	100	100	—	—	—	—	Unable to collect	Resolved or healed
	60	56	67	100	100	90	0.42	0.80	—	Stump wound closed
**No improvement or insufficient data**										
	6	—	—	—	—	—	—	—	Unable to collect	Limited information
	10	—	—	—	—	—	—	—	Before EQ-5D in registry	Worse
	15	—	—	—	75	—	0.88	—	Too sick to complete	Too sick to continue
	1	—	—	—	—	—	—	—	Left treatment	Patient decided to stop
	1	—	—	—	—	—	—	—	Left treatment	Patient decided to stop
	9	—	—	—	—	—	—	—	Left treatment	Transferred to nursing facility
	40	—	—	—	—	—	—	—	Not done	Had a skin graft

^a^HBO_2_: hyperbaric oxygen.

^b^VAS: visual analog scale.

^c^Not available.

### Pterygium Surgery and Facial Filler Injections

One particularly interesting application of HBO_2_ was for postoperative healing from pterygium surgery (Table 6). The mean age was 47 (SD 18.1) years, with 6 (86%) men and 1 (14%) woman, and all participants (7/7, 100%) classified as White. All cases came from a single center and were treated with 5 sessions each. One case had an unchanged QOL assessment before and after treatment but had no noted complications via notes after surgery, and 1 had worse QOL VAS scores after treatment (pretreatment score=85, posttreatment score=75), although the patient was noted to have uneventful recovery after surgery. The remaining patients (4/6, 67%) showed improvement in their QOL scores, with the overall median EQ-5D-5L VAS score increasing from 82.5 to 97.5, although this was not statistically significant (*P*=.38).

**Table 6:** Outcomes for patients with pterygium and facial filler injections.

Total HBO_2_^a^ treatments		Pretreatment EQ-5D VAS^b^	Posttreatment EQ-5D VAS	Pretreatment EQ-5D score	Posttreatment EQ-5D score	Reason for missing EQ-5D	Notes on outcome
**Pterygium surgery**							
	5	85	75	1	0.83	—^c^	Uneventful
	5	90	90	0.88	1	—	No postoperative problems
	5	90	100	0.86	1	—	No postoperative problems
	5	80	100	0.83	1	—	—
	5	70	100	0.88	1	—	—
	5	70	95	0.82	1	—	—
	5	—	—	—	—	Not done	—
**Facial filler injection**							
	6	—	—	—	—	Unable	Improved
	1	95	—	0.81	—	Postictal state	Had seizure
	5	75	85	0.82	1	—	Improvement in bruising or ischemia
	5	95	—	—	—	Not done	Necrosis improved with HBO_2_ therapy

^a^HBO_2_: hyperbaric oxygen.

^b^VAS: visual analog scale.

^c^Not available.

HBO_2_ was also used for complications due to the use of facial fillers (Table 6). The mean age was 46 (SD 14.5) years. All participants (4/4, 100%) were women, with 2 (50%) classified as White and 2 (50%) as missing. Of the 4 cases in the registry, 2 (50%) occurred after the use of Radiesse, a calcium hydroxylapatite filler, while the other 2 (50%) were unspecified. Of the 4 cases recorded, all were treated in a multiplace chamber, and 3 (75%) showed improvement, with 2 (50%) stopping prescribed treatment sessions early due to recovery. Both that stopped early were on a twice twice-daily schedule, with 1 at 2.0 ATA and another at 2.4 ATA. One did not have a result listed other than stopping early secondary to a seizure as a complication, but this patient was taken to 2.8 ATA, which increases seizure risk. The final case was treated at 2.4 ATA daily, completing the prescribed 5 cases with improvement. All 4 cases were treated within 1 day of referral.

### Non–Radiation-Related Osteonecrosis

Six cases of non–radiation-related osteonecrosis were treated (Table 7). The mean age was 58 (SD 17.1) years with 3 (50%) men and 3 (50%) women. The racial breakdown was as follows: White (n=4, 67%); more than 1 race (n=1, 17%); and missing (n=1, 17%). The median number of treatments was 30 (IQR 10-50). In general, these patients received long treatment courses. The improvements in QOL seen in most patients suggest that the treatment was generally successful.

**Table 7:** Outcomes for patients with non–radiation-related osteonecrosis^a^.

Total HBO_2_^b^	Anatomic location	Pretreatment EQ-5D VAS^c^	Posttreatment EQ-5D VAS	Pretreatment EQ-5D score	Posttreatment EQ-5D score	Reason for missing EQ-5D	Notes on outcome
20	Knee	—^d^	—	—	—	Not asked	—
60	Ankle	30	80	0.73	0.83	—	—
20	Lower leg	60	80	0.15	0.54	—	Uneventful course
60	Left knee	60	65	—	—	Only VAS	—
40	Jaw	35	50	0.23	0.57	—	Uneventful course
3	Sinuses or palate	50	—	0.76	—	Transferred before completion	Had lymphoma on bone biopsy, HBO_2_ suspended

^a^Currently, no detailed outcome measures specific to osteonecrosis exist in the registry. The increase in the EQ-5D suggests improvement.

^b^HBO_2_: hyperbaric oxygen.

^c^VAS: visual analog scale.

^d^Not available.

### Infected Implanted Hardware

Infected hardware was treated in 5 patients. The mean age was 49 (SD 9.6) years with 1 (20%) man and 4 (80%) women; 3 (60%) were White, and 2 (40%) were missing a racial classification. A total of 2 (40%) cases had knee hardware infection, and 2 (40%) cases had spinal hardware infection. All were prescribed HBO_2_ at 2.4 ATA for 10 to 40 treatments, but none completed >30% of their course for various reasons, including patient decision, transfer to a different facility, and significant visual disturbances. None of these patients had pre- and post-QOL measurements.

### Hypospadias

HBO_2_ was also used in the setting of hypospadias repair. Repairs of hypospadias can lead to failed grafts with scarring, fistulas, strictures, and potential deformities [14,15]. Some centers use HBO_2_ before and after the repair to improve graft and surgical outcomes. A total of 7 cases were entered into the registry. The mean age was 3.7 (SD 2.6) years. The racial breakdown was as follows: Asian (n=2, 29%); Black (n=1, 14%); White (n=3, 43%); and missing (n=1, 14%). For 5 (71%), the graft was preserved and viable, while for 1 (14%), the graft was preserved and partially viable and 1 (14%) was missing outcome data.

### HBO2 for PCC

**Figure 6:**
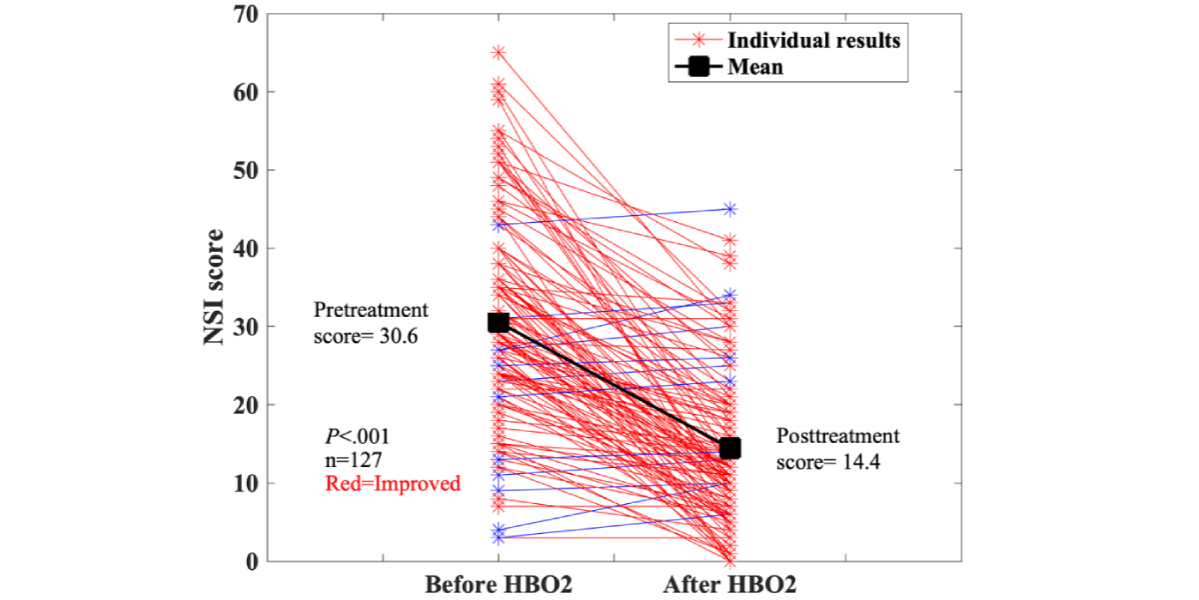
Results from the Neurobehavioral Symptom Inventory (NSI) for patients treated for post–COVID-19 condition. In the sensitivity analyses, the best (133/149, 89.3% improved; *P*<.001), worst (111/149, 74.5% improved; *P*<.001), and average (131/149, 87.9% improved; *P*<.001) cases all showed significant improvement.

A new application of HBO_2_ was for PCC. All (n=141, 94.6%) but 8 (5.4%) of the 149 cases were reported by a single center, which has an interest in treating PCC with HBO_2_ [16]. Complete pre- and post-HBO_2_ NSI data were available on 127 (85.2%) patients (Figure 6). The NSI showed a significant improvement (pretreatment score=30.6, posttreatment score=14.4; *P*<.001). Complete EQ-5D data were available on 55 (36.9%) individuals. The EQ-5D VAS also improved significantly (pretreatment score=33.6, posttreatment score=64.1; *P*<.001). The group included 80 (53.7%) women and 69 (46.3%) men (total n=149); 5.4% (8/149) of patients were Asian, 0.7% (1/149) Black, 91.9% (136/149) White, 1.3% (2/149) more than 1 race and 1.3% (2/149) were missing data on race. The median number of HBO_2_ treatments was 15 (11.5-18.5). The sensitivity analyses results are as follows: the best case (133/149, 89.3% improved; *P*<.001), worst case (111/149, 74.5% improved; *P*<.001), and average case (131/149, 87.9% improved; *P*<.001). For the EQ-5D, the cases are as follows: the best case (142/149, 95.3% improved; *P*<.001 toward improvement), worst case (49/149, 32.9% improved; *P*<.001 towards worsening), and average case (130/149, 87.2% improved; *P*<.001 toward improvement).

### Other Diagnoses

For the other diagnoses, 1 group of indications was related to the relief of hypoxia (eg, ischemic bowel, Raynaud syndrome, nonarteritic anterior ischemic optic neuropathy, central retinal vein occlusion, femoral head necrosis, branch retinal artery occlusion, vasculitic ulcer). The data on chronic anal fissures and Raynaud add to the existing case series for these conditions [17,18]. Several cases focused on the treatment of cystitis (eg, human polyomavirus 1 virus cystitis, cyclophosphamide cystitis) likely motivated by the use of HBO_2_ for radiation cystitis treatment [7]. Not enough information exists to come to conclusions about the effectiveness of the treatment for these individual cases, but this likely will change as the registry matures.

## Discussion

### Principal Findings

The registry shows how HBO_2_ is being used across a range of medical specialties for indications where hypoxia, inflammation, or both are significant factors. The emerging diagnosis treated most across the registry centers was IBD, primarily UC and Crohn disease. For Crohn disease, there were sufficient entries to show statistically significant patient-reported improvement that remained significant even with the worst-case scenario sensitivity analysis. Overall, the newly described PCC was the most commonly treated diagnosis in this cohort, although the results were predominantly from a single center. The patient-reported results showed improvement, which supports the need for further work in this area. The top 5 indications also included calciphylaxis and frostbite, which are both conditions where hypoxia is a significant consideration and where existing treatments are often inadequate.

The results also showed indications that were concentrated at particular centers, such as the use of HBO_2_ after complications for facial filler injections or to improve outcomes with pterygium surgery. The number of patients for each of these indications is limited but indicates that they could be studied in more detail to determine whether this approach should be adopted more widely at other centers.

### Comparison to Prior Work

#### IBD and Related Conditions

Several recent reviews have also concluded that HBO_2_ could be useful for IBD [19-24]. The registry data on Crohn disease support other studies showing improvement with HBO_2_ [19,25-29]. For fistulizing Crohn disease, a prospective trial has shown reduced disease activity, reduced drainage, and clinical remission when HBO_2_ is used in refractory cases [26]. The improvement in the registry of Crohn cases is noteworthy because Crohn disease is not currently a UHMS-approved indication for HBO_2_, and patients are almost always referred because standard treatments have not been successful. The patients reported significant improvement in QOL and reduced fistula and rectal discharge. Five patients had their fistulas closed. The QOL results were significant even with the worst-case approach to the sensitivity analysis. The fact that 5 patients were able to close fistulas is significant because these were likely refractory patients. Taken together, the results add to a growing set of case series and case studies that show improvement in difficult Crohn cases and support the need for large-scale trials.

For UC, HBO_2_ therapy studies have shown clinical remission, avoidance of colectomy, or progression to second-line therapy in a randomized sham-controlled trial [30]. A follow-on phase 2B trial examined the dosing strategies and concluded that 5 sessions were superior to 3 for a UC flare [31]. Typically, HBO_2_ is used adjunctively in UC with other treatments (steroids, biological agents, etc), although in 1 case study, HBO_2_ was used successfully as monotherapy for UC in an individual who had contraindications to most standard treatments [32]. HBO_2_ can be particularly useful in situations where the individual cannot receive standard treatment due to allergy, antibodies, shingles, or other reasons. The most common application of HBO_2_ in IBD is patients admitted for an acute UC flare who are at risk for colectomy or who cannot receive steroids or biological agents or where there is a delay in being able to start those agents [30,31]. In addition, patients admitted for an acute UC flare who need a bridge between when IV steroids are begun and when biological agents (ie, infliximab) start to have an effect may benefit from HBO_2_. These registry results support the idea that HBO_2_ can be useful in UC but should be interpreted with caution. Many of the patients were hospitalized for treatment and were receiving intravenous steroids and other interventions. So, while the reports of improvement are encouraging, separating the effect of HBO_2_ from the effects of standard treatment is not possible in this data set. In addition, the fact that 1 center (Dartmouth-Hitchcock) accounted for many of the treated cases (roughly 75%) and had been involved in an UC clinical trial may overrepresent the interest in UC cases across the registry centers. Also, the QOL and bowel questionnaire results were not significant in the worst-case scenario of the sensitivity analysis. Randomized controlled trials are needed to determine definitively if HBO_2_ improves on standard care.

In addition to UC and Crohn disease, IBD-related conditions where hyperbaric treatment could be applied include pouchitis, nonhealing ileal pouch-anal anastomoses, and pyoderma gangrenosum [33-37]. Two published case series and a case report show relief of symptoms and decreased disease activity in pouchitis [33,34,38]. Case series and case studies also show reduced wound size in pyoderma gangrenosum [39]. The results from this study align with these conclusions, but the registry results are descriptive and do not include a control group for comparison (as is true for most case reports and case series).

The IBD results support the idea that HBO_2_ could be considered in UC flares when standard therapy, such as steroids and biologics, is inadequate or not possible. Similarly, it could be useful in fistulizing perineal Crohn disease refractory to medical and surgical treatments. Pouchitis that has not responded to antibiotics and immunomodulators as standard therapy and pyoderma gangrenosum refractory to steroids and immunologics are also potential applications of HBO_2_. IBD may be a disease process that should be evaluated as a UHMS-approved indication for HBO_2_, as accumulating evidence shows promising results.

#### Calciphylaxis

Calciphylaxis was the fourth most treated diagnosis. Calcification of small blood vessels leads to hypoxic painful wounds, which led to the use of HBO_2_ in these patients. No randomized control trials exist to guide the use of possible calciphylaxis interventions. Such trials would likely be very difficult to carry out because the disease is rare and disease severity varies widely. Also, calciphylaxis has a very high mortality. Most patients with calciphylaxis have end-stage renal disease (ESRD), and the 1-year mortality for those with ESRD and calciphylaxis is 45% to 80%; 1-year mortality is less but still significant for those without ESRD (25%-45%) [40]. On the basis of studies done to date, HBO_2_ has been recommended by subject matter experts to facilitate healing of recalcitrant calciphylactic ulcers after accounting for cost, availability, and patient tolerance of treatment [41]. Physiologically, HBO_2_ addresses the tissue hypoxia caused by the damage to small blood vessels, which is characteristic of calciphylaxis. HBO_2_ greatly increases the amount of oxygen in circulation reaching hypoxic wounds, which promotes collagen formation and stimulates angiogenesis.

The results from this study support what has been seen in prior work. A retrospective study of HBO_2_ therapy for calciphylaxis showed promising results; 34 patients received a full course of HBO_2_, with 58% (n=20) of patients improving and 32% (n=11) completely healing their wounds [42]. In this prospective registry study, 45% (9/20) of patients improved. Similar to other studies, the registry results for calciphylaxis are mixed, which is not surprising considering the nature of this disease. Affected patients often have significant medical comorbidities, and the disease has a high morbidity and mortality rate secondary to sepsis. For example, in this cohort, 2 patients treated died of their disease, and 2 others were too sick to continue.

Nevertheless, there were several cases that improved significantly, and these successful cases had more sessions of therapy. Given the severe mortality and morbidity associated with the disease and the difficult course of standard care, HBO_2_ is a treatment that has little risk with the potential for significant benefit. The results support previous positive case series and suggest that trials on HBO_2_ and calciphylaxis are needed, and more research should be made to pursue calciphylaxis as a possible UHMS indication.

#### Frostbite

Frostbite causes hypoxia and necrosis in affected tissues, and HBO_2_ has been considered as a potential therapy for many years. Kemper et al [43] summarized the literature on frostbite and HBO_2_ in 2014. In 2021, Magnan et al [44] published a multicenter prospective single-arm study of individuals with stage 3 or 4 frostbite who received HBO_2_ in addition to iloprost (n=28). The results were compared with a historical cohort that received iloprost alone (n=30). The results showed a significantly higher proportion of preserved frostbitten segments in the HBO_2_+iloprost group. The registry results support the results from these previous studies. Most individuals with EQ-5D data reported an improved QOL. In the sensitivity analysis, this was significant in the best and average cases but not the worst case. Determining whether HBO_2_ offers an additional benefit over standard treatment is not possible within the current registry design, but future expansion efforts could include trying to include registry centers without HBO_2_ that treat similar cases.

#### Peripheral Vascular Disease–Related Wounds

Many of the emerging indications shared the underlying problem of tissue hypoxia. For the same reason that HBO_2_ therapy would work in treating acute arterial insufficiency, it might assist in the healing of chronic peripheral arterial disease ulcers. A guideline from a wound care advisory panel for the treatment of arterial insufficiency ulcers recommends adjuvant HBO_2_ for patients with nonreconstructable anatomy or whose ulcer is not healing despite revascularization. The guideline also recommends determining if the hypoxia is reversible by HBO_2_ therapy [45].

The registry cases show that some people did respond. Successful cases had at least 30 treatment sessions, while those with <15 sessions generally did not improve. Detailed information regarding each case is not available within the registry, so it is not known whether those who improved did so because they received more treatments or if they received more treatments because they had fewer comorbidities or were showing early benefits. In addition, whether the patients were screened using in-chamber transcutaneous oxygen measurements to document a local tissue increase in oxygen levels with hyperbaric oxygenation is not recorded. Patient selection is probably critical for deriving the most benefit from HBO_2_ in these patients because those with very severe hypoxia and vascular compromise may not respond. Further studies are needed to define how to select patients with peripheral vascular disease that might benefit from HBO_2_. In addition, information on screening procedures used to select these patients for treatment should be added to the registry.

#### Other Applications

An interesting application of HBO_2_ that was concentrated at 1 center, was the use of HBO_2_ after pterygium surgery. The recurrence rate of simple surgical excision can be as high as 40% but can be lowered to 16% with a conjunctival autograft procedure, although the recurrence rates are increased if the pterygium is already recurrent when the procedure is used [46]. The use of postoperative HBO_2_ treatment is based on a prospective trial published in 2011, which showed low recurrence rates when patients with pterygium underwent surgery with adjunctive HBO_2_ afterward [47]. A short 5-session HBO_2_ prescription was used. There were no complications noted with HBO_2_ in these cases and, importantly, no reported recurrences of pterygium. This local protocol may be feasible to attempt at other centers, should collaboration with ophthalmology be obtained. At the very least, this is an interesting condition to further evaluate for using HBO_2_ as a successful adjunct treatment to standard therapy. Another indication that is also focused on improving outcomes after surgery is the use of HBO_2_ for individuals who have had hypospadias repairs. There were 7 individuals in the registry with this indication, and all reported preservation of the graft, although in 1, the graft was partially viable. These results are consistent with prior work [14]. In addition, this application could be considered as an extension of the approved UHMS indication of compromised flaps and grafts.

Facial filler complications present an emergency treatment opportunity well suited for HBO_2_ and have been reported in several case reports [48-51]. This was identified in the registry cases as well. The experience of providers performing these outpatient facial filler procedures varies widely as does the ability and knowledge to address the complications. With rising interest in esthetic therapy, complications from these procedures are becoming more common. Facial fillers typically refer to hyaluronic acid fillers or particulate-based fillers, both of which are used for facial remodeling or tissue augmentation for cosmetic or reconstructive purposes. These injections can cause vascular obstruction and subsequent tissue ischemia; thus, the goal of treatment is to restore perfusion to the affected area. Although hyaluronic acid can be reversed with hyaluronidase, particulate injections such as calcium hydroxylapatite cannot easily be broken down, and HBO_2_ may play an even greater role in these cases. Expert consensus protocols include HBO_2_ in treatment algorithms for these complications [52]. Similar to acute arterial ischemic cases, success likely hinges on the hyperbaric medicine consult occurring close to the initial injury. Early intervention must be encouraged, as with other UHMS-approved indications where acute ischemia is prominent such as central retinal artery occlusion or compromised flaps, because earlier intervention leads to improved results. Half of the cases recorded were treated on the same day as the consult, although information on time elapsed since filler injection is not available. Ultimately, HBO_2_ can support threatened tissue but cannot resuscitate dead tissue, and aggressive intervention and resuscitation should be advocated. Information about the use of HBO_2_ for this indication needs to be available to other centers that administer facial filler injections.

When implanted hardware is infected, the medical standard of care typically calls for hardware removal. Removal from certain areas of the body (eg, brain and spine) can be difficult or involve complicated surgical intervention. In these instances, salvage of hardware has the lowest risk-to-benefit ratio or may be the only viable option [53]. Ridding foreign bodies of infection in vivo is notoriously difficult, which is why HBO_2_ was likely attempted in these cases. None of the patients treated completed their course of treatment, which may suggest that these cases were refractory. The results suggest that the registry should be revised to collect more detailed information about these cases.

The newly recognized PCC was treated multiple times in the registry. The anti-inflammatory effects of HBO_2_ are thought to be the mechanism of action underlying improvement. PCC is not yet completely understood but likely results from a dysregulated inflammatory process within the brain [54]. In general, patients reported improvement with treatment. This matches reports coming from case reports and series, as well as from a randomized control trial out of Israel [55]. The results need to be interpreted with caution, as placebo effects with HBO_2_ are common. Nevertheless, the data support further sham-controlled trials and suggest that HBO_2_ could be used to treat other postinfectious syndromes in the future.

Many of the other individual case reports within the registry are for indications where hypoxia is believed to be a significant factor. For example, femoral head necrosis results from a compromised vascular supply, and healing of chronic anal fissures is believed to be hampered by local hypoxia [56]. Ophthalmological conditions such as branch retinal artery occlusion, retinal vein occlusion, and nonarteritic anterior ischemic neuropathy involve hypoxia and a compromised blood supply. Raynaud syndrome and vasculitic ulcers are also characterized by local hypoxia. Currently, not enough data exist within the registry to draw conclusions about outcomes for these various conditions, but over time, additional cases will accumulate.

The hyperbaric registry is still in its nascent stages. Greater adoption of the registry by the community will lead to more significant results. The cases highlighted in the report have identified additional data that should be recorded for some of these indications. For example, facial filler cases highlight the need for further information such as the time of injury to help determine the optimal timing for maximal HBO_2_ efficacy. In addition, the facial filler cases should be documented in an identified category, as is done with indications such as UC or Crohn disease, rather than just marked as “other.” More specific outcome measures are needed for non-radiation–related osteonecrosis cases. In addition, long-term follow-up will be essential for infected hardware cases because the outcome may not be known until well after the treatments have stopped.

Many of the involved centers are academic, nonprofit, and governmental entities with an interest in advancing evidence-based medicine and research. The contributions made by such centers are essential to evaluating the promise of HBO_2_ in various conditions and may lead to the adoption of them as new UHMS indications. This review provides a snapshot to clinicians of the diagnoses that have been treated by HBO_2_ that are not officially on the UHMS indications list. This can be useful in situations where patients are not improving with standard therapy, and other approaches are being sought that could address hypoxia and inflammation. It also focuses attention on specific diagnoses worthy of further study.

### Limitations and Strengths of This Study

The registry has limitations. To keep the time required for data entry to a minimum, only a specific set of outcomes is collected. The registry does not include extensive data on medications, other comorbid conditions, or laboratory results. While this approach has been successful at making the registry practical for centers to adopt, it does affect the scope of conclusions that can be drawn.

The registry undergoes active review and expansion over time, so not all questionnaires currently in use existed in the registry when it started. Missing data, particularly for the EQ-5D in earlier cases, is understandable, as this questionnaire was added later in registry development. In addition, data collection can be missed if patients stop treatment early or unexpectedly or transfer to another center. Nevertheless, the missing data are a limitation of the registry. The registry does not include a control or standard treatment group for every condition, so it is not possible to compare outcomes with no treatment or placebo. Registry results can be compared with publicly reported treatment success rates of those using standard therapy.

For the analyses, the data were reviewed to fix indications that were misclassified (ie, UHMS-approved indications that were categorized as “other” or “other” indications that were inappropriately classified as UHMS-approved indications). There may be cases, however, that were missed. The data are dependent on data entry, and there may be variations in data entry quality between centers. Work is ongoing with data quality checks throughout the registry centers to ensure that these problems are limited in the future.

Nevertheless, the strength of the registry is that it allows outcomes from less common conditions to be combined across centers, which provides information that no individual center would be able to achieve independently. The accumulated results identify areas where more detailed trials would be useful. In addition, some centers are beginning long-term follow-up with treated patients to gather data on satisfaction with and longevity of the results. This will expand the capabilities of the registry although the responses will be affected by selection bias.

### Conclusions

The registry is providing standardized outcome data on conditions that are not currently UHMS approved but are being treated with HBO_2_ at participating centers. This is adding prospectively collected data to the mostly retrospective data that exist in the literature. Results show statistically significant improvements in patient-reported outcomes, such as QOL, for IBD, frostbite, and PCC. HBO_2_ is also being used to improve surgical outcomes for pterygium and hypospadias procedures and to treat complications from facial filler injections. HBO_2_ is also being tried for difficult cases involving hypoxia such as femoral head necrosis, Raynaud syndrome, and chronic anal fissures.

As time goes on and as more centers participate, significant trends can be identified, which may identify a valuable treatment option for medical conditions where hypoxia and inflammation are important contributing factors. Indications developed at a few centers initially can also be expanded to other centers based on the outcomes.

## Appendix

Multimedia Appendix 1 Perianal Crohn Symptom Index.

Multimedia Appendix 2 Bowel Symptoms Questionnaire.

## Abbreviations

ATA: atmospheres absolute
ESRD: end-stage renal disease
HBO2: hyperbaric oxygen
IBD: inflammatory bowel disease
IRB: institutional review board
NSI: Neurobehavioral Symptom Inventory
PCC: post–COVID-19 condition
QOL: quality of life
RECORD: Reporting Of Studies Conducted Using Observational Routinely Collected Health Data
REDCap: Research Electronic Data Capture
STROBE: Strengthening the Reporting of Observational Studies in Epidemiology
UC: ulcerative colitis
UHMS: Undersea and Hyperbaric Medical Society
VAS: visual analog scale

## Data Availability

The data and code used for the study are available from the Steering Committee of the Multicenter Registry for Hyperbaric Oxygen Treatment upon reasonable request.
