# Supplementary material for: Emerging Indications for Hyperbaric Oxygen Treatment: Registry Cohort Study
Source: Interact J Med Res. 2024 Aug 20;13:e53821. doi: 10.2196/53821 (PMC11372337; doi:10.2196/53821)
Supplement: Multimedia Appendix 1 [file ijmr_v13i1e53821_app1.pdf]

Date Completed____________ REDCap #_____________ Pre____ Post____ Long Term Follow-up ______ months

PERIANAL CROHN'S INDEX

1. **Do you have an anal fissure or tear in the anal area that won't heal?**

Yes	No (if no skip to question 3)

2. **How much of a problem is this anal fissure or tear**

None	A little problem, occasional pain and discomfort	A medium problem with pain and discomfort regularly that's manageable	A major problem with significant pain and discomfort that affects my life

3. **Do you have a fistula or fistulas (pathway from the bowel to the skin or other organ) in/around the anus? If yes, how many?**

No (skip to number 5)	1	2	3	>3

4. **In total, how much discharge do you have from the fistula(s)?**

None	At little bit of mucus	A medium amount of mucus and pus	A lot of mucus and/or pus	Stool/feces coming through

5. **Do you have a rectal discharge?**

No discharge	A little bit of mucous discharge	A medium amount of mucous or pus discharge	A lot of discharge	Gross fecal/stool soiling

6. **Are there areas in your anal region that are swollen, firm, or hard to the touch? If yes, how many?**

No	1	2	3	>3

7. **Do you have setons in place? If yes, how many?**

No	1	2	3	>3

8. **Bowel Frequency/Number of Bowel Movements – Daytime**

1 - 3	4 – 6	7 – 9	>9

9. **Bowel Frequency/Number of Bowel Movements – Nighttime**

0	1 - 3	4 - 6	>6

10. **Urgency of Defecation/Feeling that Need to Move Bowels Right Away**

None	Need to Hurry	Need to Move Bowels Immediately	Incontinence/Can't Get to Toilet in Time

11. **Taking Diphenoxylate/atropine (Lomotil), Loperamide (Imodium) or Opiates for Diarrhea?**

No______

Yes______ Frequency of Meds

1-2 days/wk	3-4 days/wk	5-6 days/wk	daily

12. **Visible Blood in Stool?**

None	Small streaks of blood	Occasionally bloody	Usually bloody

13. **Abdominal Pain/Pain in Stomach?**

None	Before/after bowel movements	Prolonged

14. **Taking medications for abdominal pain/pain in stomach?**

No______

Yes______ Frequency of Meds

1-2 days/wk	3-4 days/wk	5-6 days/wk	daily
