# Supplementary material for: Emerging Indications for Hyperbaric Oxygen Treatment: Registry Cohort Study
Source: Interact J Med Res. 2024 Aug 20;13:e53821. doi: 10.2196/53821 (PMC11372337; doi:10.2196/53821)
Supplement: Multimedia Appendix 2 [file ijmr_v13i1e53821_app2.pdf]

Date Completed____________ REDCap #_____________ Pre____ Post____ Long Term Follow-up ______ months

Bowel Symptoms Questionnaire

Before you began experiencing bowel problems, how many bowel movements did you typically have in a 24 hour period? [ ]

In the last 24 hours how many bowel movements did you have? [ ]

How many times do you experience bowel movements during the day?	0 - 6	7-10	11-14	15 - 19	20+
How many times do you experience bowel movements at night?	0	1	2	3	4+
If you get up to go to the bathroom at night, does it bother you?	Never	Mildly	Moderately	Severely	
Do you have blood in your stool?	Never	Occasionally	Usually	Always	
Do you have pain associated with your bowel or in your pelvis (rectum, GI tract, etc.)?	Never	Occasionally	Usually	Always	
If you have pain with bowel movements, is it usually…		Mild	Moderate	Severe	
Does your pain bother you?	Never	Occasionally	Usually	Always	
Do you have urgency associated with the need to move your bowels (feeling the need to move bowels right away)?	Never	Occasionally	Usually	Always	
If you have urgency to move your bowels, is it usually…		Mild	Moderate	Severe	
Does your urgency bother you?	Never	Occasionally	Usually	Always	
